# The True Uterine Mucous Membrane, Its Structure, Functions, and Morbid States

**Published:** 1875-05

**Authors:** E. N. Chapman

**Affiliations:** Brooklyn, N. Y.; Late Professor of Obstetrics, etc., L. I. College Hospital


BUFFALO

Medical and Surgical Journal.

VOL XIV. MAY, 1875. No. 10.

### Original Communications.

:o:

ART. I.—*The True Uterine Mucous Membrane, its Structure, Function and Morbid States.* By E. N. CHAPMAN, M. D., late Professor of Obstetrics, etc., L. I. College Hospital.

To arrive at a just appreciation of the diverse states of the true uterine mucous membrane in health and disease, it is necessary to study these from a physiological, a histological, and a clinical standpoint. By this precaution against illusions, the view presented by one will correct the others, and the conclusion formed from all be a near approximation to the truth, the nearest possible to an observer, dealing with vital, ever-shifting phenomena. To assume at the start that all mucous membranes have a like structure, perform a like office, and suffer like morbid changes, and then, on the basis of such data, to settle the whole matter in dispute by logical inferences, can but lead to grave errors, so grave as to block all real progress, and render treatment inefficient, if not detrimental. True, the gastric, rectal, vesical, vaginal, cervical and corporeal mucous membranes have a general resemblance, a common ground-work, and yet each has a special structure, function, and pathology—a something over and above what belongs to its fellows- -which is not to be appreciated fully except by the light shed upon them by clinical facts. That there is a radical difference is obvious to the least critical observer, but in what this difference consists, it is scarcely possible, with all the helps afforded to medicine by modern science, to decide. The microscope, though it reveals the myriad little workers that build up and take down this living fabric, yet fails to wrest the secret from unwilling nature. The rectum cannot fill the place of the stomach, the vagina of the bladder, or the cervix of the corpus uteri, nor can the normal or morbid states of one part be predicated from those of an other. Each has an individuality even where the likeness is nearly complete, as in the case of the vagina and rectum, but in the case of the corporeal mucous membranes, the theatre of such unique evolutions at puberty and the menopause, and during menstruation and utero-gestation, this individuality is striking and unmistakable. Because the exudation of coagulable lymph and the formation of a false membrane may, as a result of inflammation, take place in the trachea, it does not follow, as first taught by William Hunter, 1774, that the decidua vera must be a like process. Nevertheless this doctrine, so contrary to all known vital phenomena, held sway until called in question by Sharpey, 1837. Following him, E. H. Weber, Coste, Goodsir, Robin, et al., have conclusively demonstrated that the decidua is nothing more or less than the hypertrophied and transformed mucous membrane, which, on the uterus being freed of its burden, either timely or untimely, degenerates, breaks down, and comes away in the lochial discharge. The underlying muscular walls being thus denuded, it is, according to Kolliker and Simpson, from six to ten weeks before the new membrane is fully formed. The death of the old and the birth of the new, is so unlike any thing else met with in the animal economy, unless it be the restoration of lost parts in the lower orders, as to show beyond question that here is a tissue *sui generis*, one demanding for its elucidation special study.

The membrane, thrown off in a certain species of dysmenorrhœa, has, until a recent date, been regarded as effused and organized coagulable lymph, but whether the decidua of a blighted ovum or the product of disease, simply was undetermined. "It is composed of plastic lymph (such as we see secreted by the mucous membrane of the trachea in croup) thrown off by the lining membrane of the uterus, and taking generally the form of the cavity of that organ, although it may be discharged in shreds."—Churchill. "Fibrin is poured out on the internal surface of the cavity of the uterus, and assumes a membranous texture, as we find occurring in other hollow organs lined with a mucous membrane, as, for instance, in the intestines in diarrhœa tubularis, and in the trachea and air-tubes."—Montgomery. "That this membrane is produced by a similar state of inflammatory action to that which some times occurs in other mucous surfaces and give rise to a similar exudation, is most probable notwithstanding the absence of general inflammatory phenomena, and the neuralgic character of the pain."—Copland.

The earliest description of this membrane was given by Morgagni, 1723, who thus describes it as met with in the case of a "noble matron" that had "pains like those of childbirth." "In almost the middle of the menstrual flux, a membranous body, as it appeared, was discharged from the uterus, and that in such a form, and such a magnitude, as perfectly corresponded to the triangular form of the uterus, being moderately convex externally; on which surface it was unequal and not without many filaments that seemed to have been broken off from the parts to which they had adhered, but internally hollow; on which surface it was smooth and moist, as if from an aqueous humour which it had before contained, but had discharged at its own exit by an ample opening, which was at one of its angles that had been really opened by rupture." The menstrual decidua, as it has been named by Virchow, is thus represented by Denman, 1794: "Several years ago I requested the favor of Dr. Bailey to examine some portions of this membrane, and he agreed with me in thinking it an organized membrane similar in structure to the decidua." Arriving at the conclusion that this substance is not always "a consequence of early conception;" he states "that the uterus has, occasionally or constantly, in some women, the property of forming it, at or in the interval between the periods of the menstrual discharge." In 1821, John Powers used these significant words: "In women placed out of all improper suspicions, where the local actions of the uterine system are carried to a greater than customary height, the actions of the uterus preparatory to the reception of the ovum extend to an actual production of the deciduous secretion, so that the menstrual discharge becomes a *bona fide* discharge of decidua membrane."

In April, 1846, Oldham writes, "How is this membrane produced? It is generally thought to be lymph, but if some good specimens are carefully examined they will be found to possess the same structural elements as the uterine decidua, * * * they are full of little holes with epithelial scales, which I cannot doubt are openings and epithelium of the follicles of the uterine glands."

In September, 1846, Simpson showed conclusively that the dysmenorrhœal membrane is composed of the same anatomical elements as the decidua vera, and formed by a similar physiological process. He states: "The proper mucous tissue of the uterus itself may within the compass of a menstrual period, form, enlarge, separate, and again be reproduced; and further, that all this may occur and continue regularly for a succession of months, or, as some times happens, for a succession of years. Essentially, the normal action of the uterus or ovaries giving rise to the formation of the dysmenorrhœal membrane, is not a state identical with inflammation, but a state identical with the condition of these organs after impregnation and during the earlier weeks of pregnancy." The truth of the above doctrine that was soon after verified by Hanfield Jones, Lebert, Raciborski, Virchow, Kolliker, Scanzoni and others, is now settled as far as any physiological fact can be. Scanzoni says: "A minute examination of these membranes, which we have made at various times, in concert with Kolliker, has convinced us that they are nothing else than the hypertrophied mucous membrane peeled off from the internal surface of the uterus. The formation of these membranes, the histological texture of which offers a great analogy with the decidua which is formed after conception, is occasioned without doubt by a considerable and often repeated hyperæmia of the walls of the uterus, which is followed by an excess in the development of the mucous membrane."

The decidua vera and the dysmenorrhœal membrane being thus conclusively proven to be the true uterine mucous membrane, hypertrophied and exfoliated, it becomes a question of interest and importance, what changes, if any, take place in it, as a result of the menstrual act under other conditions.

The corporeal mucous membrane is as unlike either the cervical or the fallopian as it is any other found in the body. It terminates abruptly at the inner os uteri, and the uterine orifices of the fallopian tubes, and alone takes an active part in the evolutions of menstruation and pregnancy. It has no submucous or interstitial areolar tissue, "but only a small quantity of spindle-shaped fibro-plastic fibres, scattered between the tubules."—Dalton. Its "basement layer is that connective tissue which is seen throughout the female genital organs, containing undeveloped nuclei and fibre-cells, but no elastic elements."—Kolliker. "Besides utricular glands, capillary vessels and epithelium, it is formed of free elementary corpuscles or nuclei, contractile fibre cells and amorphous connective tissue."—Farre. The utricular glands compose the bulk of the membrane, and taking a tortuous, sprial course open on the free surface of the mucosa. Slender capillaries pass up from the muscular tissue along the sides of the glands and surround their mouths with a net work of vessels. Ciliated epithelium covers the mucosa and lines the glands which "in their normal condition contain no morphological particles; but their epithelium is very easily detached, and may appear as a whitish-gray secretion filling them."—Kolliker. The mucosa, ordinarily "white or reddish-white," presents at the periods "a phlogosed and turgescent appearance," and in cercain subjects "a more or less violet-hue." "The vascular apparatus of the womb is coincidently developed, and injected in an extraordinary manner; that of the mucous membrane especially, forms at the surface of the membrane beneath the delicate layer of epithelium with which it is invested, an elegant net work with irregularly lozenge-shaped meshes, each of which incloses the orifice of one of the innumerable glandular tubes of which it almost wholly consists "Upon inspecting the extraordinary thickness of this membrane, one might be led to suppose it the seat of a true pathological hypertrophy, or other alterations, were it not that repeated experience, corroborated by an examination of the parts, in women dying of accidents at the commencement of pregnancy, afforded us the undeniable proofs of its being a normal condition of things."—Coste. (Meigs' Obstetrics 5th Ed.) These histological changes, as above represented, are confirmed by Kundrat, (*Med. Jahr.* Vol. 11, 1873, and *Med. Times and Gazette,* July 26, 1873,) who has made an extended series of microscopical examinations of the corporeal mucous membrane before, during, and after menstruation, and in the interval, but the order of these changes is so reversed, and the relation of one to another so disturbed as to throw more than doubt on the ovular theory and the inferences drawn therefrom. According to Kundrat, the vascularity and swelling of the mucosa attain their height "several days" preceding the flow, by which means the uterus is prepared a certian time in advance of the rupture of the Graafian vesicle for the reception of the ovum. This not being fertilized, the mucosa which is thick and loose from the elongation and dilatation of its glands, and growth of its inter-glandular matrix, suffers fatty degeneration of its more superficial layers. This retrograde process, homologous to that occurring at the termination of pregnancy, causes the flow, by the capillary vessels and cells of the stroma being involved as well as the epithelium. The anatomical sequence is "swelling of the mucosa, fatty change in cells and vessels, vascular rupture and hemorrhage." The flow being over, it is a short time—a day or two—before the mucosa has returned to a state of rest. The type of the impregnated uterus is seen in the active uterus when the mucosa is swollen and menstruation has not yet commenced.

The ovum being fertilized the mucosa enters on a higher stage of development that it may elaborate the pabulum for the plant-like growth which, by tiny roots, is to draw its sustenance from the albuminoid secretion of the uterine glands.

Dr. John Williams, Assistant Obstretric Physician to University College Hospital, London, presented at a resent meeting of the Royal Society of London, a series of observations made on the uteri of nine women who had died in different stages of the monthly period. In two of the uteri the menstrual flow had almost ceased, and the mucous membrane was wanting in the bodies of the organs. The muscular fibre-cells were more or less exposed in the cavity, and the meshes formed by their bundles contained glands and groups of round cells.

In one uterus menstruation had ceased three days before death, and the muscular fibres were not exposed in the cavity of the organ, but imposed upon them was a layer of tissue composed of fusiform and round cells. This tissue contained glands. The muscular tissue near the internal orifice was devoid of glands, but nearer the fundus it contained numerous glands.

In one uterus, in which the catamenial flow had ceased probably about a fortnight before death, the layer of superficial tissue was thicker than in the last; and near the internal orifice there was a marked and abrupt distinction between it and the subjacent muscular tissue.

In one uterus the flow had ceased three weeks before death, and the superficial layer was still thicker; and the distinction between it and the subjacent muscular layer was well marked, except at the fundus. The uterine glands were tubular, and arranged in some parts obliquely, in others perpendicularly to the surface. They were lined by columnar ciliated epithelium.

In two uteri menstruation was imminent, but the flow had not begun. In these the mucous membrane of the body of the uterus was fully developed, and had begun to undergo fatty degeneration. There was a marked distinction between it and the muscular tissue throughout the uterine cavity; it was highly conjested.

In one uterus the menstrual flow had taken place for one day, and in another for two or three days before death. In these there was extravasation of blood into the mucous membrane, and the latter had in part been disintegrated and removed.

Menstruation appears essentially to consist, not in a conjestion or a species of erection, but in growth and rapid decay of the mucous membrane. The menstrual discharge consists chiefly of blood and of the débris of the mucous membrane of the body of the uterus. The source of the hemorrhage is the vessels of the uterus. The mucous membrane having undergone fatty degeneration, blood becomes extravasated into its substance; then the membrane undergoes rapid disintegration, and is entirely carried away with the menstrual discharge. A new mucous membrane is then developed by proliferation of the inner layer of the uterine wall, the muscular tissue producing fusiform cells, and the groups of round cells enclosed in the meshes of the muscular bundles producing the columnar epithelium of the glands.—*Obstetrical Journal of Great Britain*, August, 1874.

It thus appears that a nest is being prepared in the uterus for the reception of the egg before it leaves the ovary, and that, on the failure of conception, a new one replaced the old, month after month, and year after year, until the vitality of the genitalia is expended, and the menstrual involution terminates in the climacteric. Hence the activity of the arteries, the fullness of the erectile vessels, and growth of the mucosa, being the preliminary stage to an actual incubation, are collectively embraced in the term menstrual nidus which the writer is wont to use as better representing than any other the sum of the phenomena presented. If vitality be imparted to the ovum, the stimulus to a higher development will exist, but if not the same metamorphosis will take place as after labor and abortion. Each monthly discharge is a sign of a "baulked or disappointed pregnancy" the condition of the uterus on the appearance of the ovum, whether impregnated or not, being identical.

The nature of the menstrual decidua may be further studied by observing the more remarkable transformations of the mucous membrane during pregnancy. The facts, though wrongly interpreted, are well described by William Hunter, in his great work, The Anatomical Description of the Human Gravi Uterus and its Contents, published in 1794. "This membrane (the decidua) is an efflorescence of the internal coat of the uterus itself, and is, therefore, shed as often as a woman bears a child or suffers a miscarriage. It is of considerable thickness, and one stratum of it is always left upon the uterus after delivery, most of which dissolves and comes away with the lochia." At about the sixth week of pregnancy "the decidua is observed to be readily separable into irregular portions or fragments, with clear interspaces—very much, in fact, like a web or network—founded by the superposition of several layers of a cribriform membrane one upon another"—Priestley.

The decidua reflexa is formed, according to the view of William Hunter, by the ovum as it enters the uterus thrusting a portion of the decidua vera before it, and of Sharpey and Coste by the decidua vera at the place of lodgement of the ovum growing up around it like granulations around an issue pea. This process, however, which is strictly vital and has nothing akin to mechanical force or inflammatory action is better represented by Farre who claims that the ovum "drops into one of the orifices leading to the utricular follicles, and in growing there draws around it the already formed but soft and spongy decidua constituting the walls of the cavity;" or by Weber who asserts that, on the ovum reaching the uterine cavity, the decidua reflexa is first formed by the development of a superficial layer of the mucous membrane, and then the decidua vera by a like change in a deeper layer. These two layers remain united at the point destined to be the site of the placenta.

It seems evident, therefore, that preceding the rupture of the Graafian vesicle the most superficial layer of the mucosa will be in a state of preparation to become either the decidua reflexa or the decidua menstrualis, according as conception shall or shall not occur, but that the deeper layers will only be transformed into the decidua vera by the stimulus of the growing embryo. This impulse being present, the entire mucous membrane is converted into laminæ which on the absorption of the nutritive fluid thrown out in their interstices and the fitness of the placenta to begin its office, are blended into one.

At the fourth or fifth month, according to Kilian, Robin, Farre and others, "a more or less soft pulpy substance which has the same anatomical composition as the decidua" is formed on the inner face of the uterus. This substance is not discharged during labor, but becoming "the seat of the reparatory process" assumes in from twenty to thirty days the character of a mucous membrane. Beneath the decidua are "found small and regular epithelial particles, not mature at the time of delivery, but embryonic in character, and probably the progeny of the original mucous membrane. The epithelial cells forming the mucous membrane of the uterus at the time of conception, were, no doubt, transformed into the fibro-cellular structure of the decidua, and hence the reproduction from the basement membrane, during the latter half of pregnancy, of these cell-particles which were intended to replace the original mucous membrane"—Priestley.

From the foregoing anatomical data it is apparent that the physiological evolutions attendant upon menstruation and conception are one and the same, the superficial layer or layers of the mucosa growing, degenerating and falling whilst the separative process is going on beneath; and that in the one case or the other an interval elapses before the parts return to their original condition, this interval being after menstruation from two to three, but after delivery from sixty to seventy days. The only real difference is this; the changes are on a smaller scale in the non-pregnant than in the pregnant uterus. So, likewise there is in each the same excitement of the arteries, repletion of the erectile vessels, and irritability of the nerves of the uterus, ovaries and vagina, save that conception intensifies and perpetuates the vital activity begun by menstruation.

The life-force lying dormant in the basement membrane is the cell-particles, cell-bodies, germ-cells or embryonic-cells. These imbedded in a matrix constitute the formation matter, the protoplasm, the living protein of which so much of late has been written by Schultze, Brucke, Kuhne, Haeckel, Huxley and others. First the wall and second the nucleus of the cell being eliminated as accessory and unimportant, there was nothing left but its contents—carbon, hydrogen, oxygen and nitrogen variously combined —which by virtue of its molecular forces constitute, it is claimed, "the physical basis or matter of life." Whether cell-growth or molecular force is the prime factor in the initiative phenomena which are ranked as vital, it is quite certain that nowhere in the human organism is the "living cell-body, or elementary organism" so abundant, so unstable, so sensitive to stimuli, so prone to start upon and run through its brief existence as that forming the matrix of the true uterine mucous membrane. A normal menstruation signalizes the destruction of the superficial cells and capillaries only, but a profuse menstruation indicates that the deeper tissue and the larger capillaries are involved. As the haemorrhage in menstruation and abortion springs from the same source and arises from the same fatty change, it follows as a matter of course that a metrorrhagia or a menorrhagia, attended with a like profuse, clotted flow must be due to a like destruction of the mucous membrane. So, likewise, in each must there be a like activity of the arteries, repletion of the veins and irritability of the nerves of the uterus and ovaries. In a word the pathological is a "counterfeit presentment" of the physiological, the one being as it were the reflected image of the other. In metrorrhagia the mucous membrane is not fully reformed so that the blood flows in fitful, copious gushes from the open mouths of the uterine vessels. In menorrhagia the mucous membrane grows too rapidly, degenerates too profoundly, falls too quickly, so that the flux is premature, excessive and prolonged. In both as after an abortion, the uterus does not involute but remains large, vascular and spongy, an abnormal state strictly analogous to the normal.

The epithelial cells of the mucosa growing and dying periodically from normal stimuli, and, from abnormal, the deeper tissues and even the entire membrane being hypertrophied and disintegrated, there can be no chronic endometritis, fungosities, or other inflammatory change, inasmuch as, should the circulation be much increased, the diseased structure, together with its morbid products would be thrown off at the next monthly period. This decadence which has been compared to "moulting" by Muller and "pregnancy on a small scale" by Virchow, lays bare the underlying muscular walls more completely and thoroughly than is possible by an escharotic or the curette, the necessity for which under such circumstances it is difficult to divine. The assumption of a chronic metritis, united with the endometritis does not help the matter as now the exuviation would be even more complete than before, and leave nothing to burn off or scrape away except the bare muscular walls and the gaping mouths of the bleeding vessels. The utricular glands are, by the menstrual nidus, prepared to secrete an albuminoid product for the nourishment of the ovum, should, perchance it be vitalized; but, on this purpose of Nature being thwarded, their epithelial cells suffer fatty degeneration with the tissues which they cover, and appear in the discharge as muco-purulent matter. This process, which in any other mucous membrane would be morbid were its tissues thus affected, is, in the uterine, the condition of prime health, and no more calls for the intervention of the utero-surgeon than the like process occurring at the premature expulsion of the fœtus.

The proliferation, or diffuse growth of connective tissue that is stated by microscopists to take place in both the mucous membrane and muscular walls of the uterus, whenever these are morbidly developed, has been claimed to be proof positive of inflammation. This word, proliferation, is made to do yeoman's service in the cause, bearing aloft the banner of the phlogistic pathologists and leading on the heroic band of operators who follow closely on their heels; and yet the tissue in the uterus called connective is peculiar to this organ and unlike that met with in other portions of the body—as much so as the menstrual congestion is unlike a pulmonary or a hepatic. This dissimilarity, broadly indicated by Dalton, Kolliker and Farre, as hitherto seen, is generally admitted by other microscopists. The growth of this singular, unique tissue, which in morbid hypertrophic conditions of the uterus is asserted to be in excess of and in disproportion to the other elements is shown by Kundrat to occur, normally, in the corporeal mucous membrane each mouth as a preliminary and a preparatory step to the menstrual act. The observations of this writer, extending over a series of cases and prosecuted under every varied condition merit the highest consideration inasmuch as they were made from an anatomical standpoint, and not disturbed by theoretical prepossessions. They throw more than doubt on the doctrine that uterine hyperplasia is due to either inflammation or ordinary congestion, and bring out into a clearer light the real significance of proliferation. Previously, it had been stated by Kolliker that on conception taking place, "the vessels of the mucous membrane are seen to be more dilated, and we may observe an abundant new formation of connective tissue in its parenchyma, while considerable enlargement of the tubular glands has also taken place." Thus here as elsewhere in the genital system the abnormal is related to the normal, the one being only a perversion of the other.

Moreover, the corporeal mucous membrane is not only unlike the others, the rectal, vesical and vaginal or even the cervical and fallopian, but is equally unlike itself at the several stadia in the life of the female. The first is the fœtal stage when the uterus advances to a certain point and there rests during childhood, the second the adolescent when, according to Arnold and Klob, it again goes forward for four, five or six years until the goal of womanhood is reached, and the third the climacteric when having finished the race it retraces its steps and yields its life at the point whence it started. Besides, at the reproductive age there are other stadia more notable still, the advance of the periods and the retrogression of the menstrual involution, and the advance of pregnancy and the retrogression of the puerperal involution.

In a state of rest the walls of the uterus are by their contractility placed in such close opposition that there is no vacant space in its body or at the os internum. The influx of blood into the venous canals relaxes its muscular fibres and makes room for the swollen mucosa at the periods and the growing embryo during pregnancy, so that in each case a cavity is formed by the vital expansion of the uterus itself and not by the dilatation of an internal force. In the normal state therefore, there can be no obstruction notwithstanding the existence of a shut cavity, and in the abnormal such obstruction when present must, almost always, be sought in defective or excessive nervous and vascular excitement of the uterus that retards, checks, or prevents the due maturation and dehiscence of the mucous membrane. This fault, and not stricture and pent up blood, is at the bottom of most cases of scanty, delayed, and absent menstruation, and the symptoms arising therefrom, and is only to be remedied by placing the forces of Nature under favorable conditions.

The vitality of the genitalia beginning and ending with the reproductive age, when the irritation of nerve-fibres, the repletion of erectile vessels, the growth of mucous tissue and the rupture of Graafian follicles occur periodically, womb disease will not, since its remote causation is always a disturbance of this function, be met with before puberty or after the menopause. The same may be said of all morbid growths, benign or malignant, which invariably originate in the period of vital activity, though undetected until later in life.

The histology, physiology and pathology of the membrane lining the inner face of the body of the womb being, by the concurrent testimony of the most reliable observers, thus definitely settled, it behooves the wise practitioner to see to it that his treatment rests on this solid foundation and not on unsubstantial theory. Analogy, induction, and reasoning have not, and never will solve the mysteries of life, nor add anything of value to the common stock of medical knowledge. Of enthusiastic, imaginative men, men of shining parts, medicine has, in times past, had enough. To-day the profession is not overproud of their record, nor is the world overmuch in their bebt, notwithstanding they, in their generation, occupied the chief places and were regarded as oracles. This remark, sweeping as it may seem, applies to such as Benjamin Rush, whose bold hypothesis were, by their general acceptance the first quarter of the present century, of infinite injury to medical science and the human family. Would it not have been far better had he been content with empirical facts, like the great modern clinical teachers, Trousseau and Niemeyer, and held the higher exercise of his reason in reserve for metaphysical subjects? As it is, many an ignorant quack has by dealing with facts alone, though blindly, left more behind him of advantage to science, and of benefit to the race than this once famous professor. This doctrine that menstruation, pregnancy and parturition were different grades of inflammation sounds singularly enough to modern ears. One cannot but wonder where he found the materials to build such an airy castle, and yet this doctrine is not a whit more irrational than those now promulgated from high places in regard to the pathology of uterine disease.

It being taken as a starting point that the genital like other organs must, sooner or later, become inflamed when long the center of irritation, the symptoms and signs of disease are so distorted as to support foregone conclusions. All ill success demanded new theories and new expedients, the seat of this inflammation has from time to time, been shifted from the cervix to the ovaries, from the ovaries to the corpus uteri, and from the corpus uteri to the mucous membrane; so that now, by a blending of these several morbid states into one, the cavity of the uterus is thought to be lined by a pyogenic membrane, surrounded by indurated walls, and obstructed by a contracted outlet, like the cavity of a deep seated abscess. These premises being granted as beyond question, it stands to reason, so the argument runs, that this long, narrow tract must, in the first place, be opened by dilatation or incision, if the real seat of the disease be reached, and that this bleeding, suppurating fungous surface must, in the second, be condensed by astringents, revolutionized by alteratives, or destroyed by escharotics, if the proper treatment be instituted. Thus, in spite of the flood of light thrown of late years upon the structure and function of the genital organs of the female, this class of gynæcologists still grope on in the old paths, either shutting their eyes to the new and better way which the microscope has opened up before them, or, at the best, blending the errors of the past with the discoveries of the present in a strange jumble which is neither one thing nor another. This veneration for old theories after their complete refutation is quite inexplicable. Hodge maintains that womb-disease is wholly due either to irritation or sedation of the genital nerves, a revival of the "irritable uterus" of Gooch, and yet admits the possibility of endometritis as an exceptional disease. In the uterine structures other than the moucous, he claims, that the nerve-disturbance never induces a state of the vascular system more serious than an ordinary hyperæmia. Klob and Atthill discard the inflammatory and neuralgic doctrines, and fix upon congestion a fulness of the blood-vessels such as is observed in other organs, as the real pathological condition, and yet, without offering any reason whatever, simply assert that there is such a disease as endometritis. The former says: "If, therefore, we are constrained to consider the process producing the menstrual decidua as an excess of menstrual phenomena, especially in the mucous membrane of the uterus, it follows, that those pathologists were not far from the truth, who described such cases as endometritis." The latter begs the question in these few words: "This dysmenorrhœal membrane is probably an exfoliation from the mucous membrane which lines the cavity of the uterus, and is most likely the result of chronic inflammation."

In 1867 Hewitt enters into a long argument to prove that inflammatory nature of uterine disease and in regard to membranous dysmenorrhœa remarks. "There is no doubt whatever that the membrane discharged in these cases is really the uterine mucous membrane, hypertrophied probably in consequence of an excessive nutritive activity in its tissues, to which without impropriety the term endo-metritis may be applied."

In 1872, however, he discards inflammation and adopts flexion as the cause of all benign, uterine disorders. This bending at the site of the os internum, as he now thinks, induces irritation by pressure on the nerves, congestion by obstructing the veins, stricture by narrowing the cervical canal, and prolapsus by increasing the weight of the uterus. He more than doubts the presence of endometritis in any case, asserts: "That this disordered state of the lining of the body of the uterus is the result of retention of natural secretions, and the irritation proceeding therefrom" and endorses the opinion of Hausmann: "That these perfect casts are really early abortions." In both editions, the second and the third, of his work abundant clinical evidence is adduced to establish either proposition.

Thomas* and Barnes adopting inflammation as the ultimate pathological state in the diverse manifestations of uterine disorders fail to see anything significant in the histological discoveries of recent times. Thomas wholly ignoring their importance says of membranous dysmenorrhœa: "We know very little with reference to etiology, course, or treatment. Our want of precise knowledge depends upon the fact that the true pathology of the condition is not settled." Barnes though conceding the importance of these discoveries maintained that the falling of the membrane is a mere epiphenomenon in the course of endometritis. Excluding the cases due to an early abortion he asserts that "Dysmenorrhœa

* The views of Prof. Thomas, as published in 1869, have since by the singular mutations to which the doctrines of gynæcologists seen very prone, undergone essential modification. He now considers the argument about inflammation and congestion a matter of words and favors the mechanical pathology as promulgated by Hewitt and others of that school.

membranacea may be classed under the inflammatory kinds" and again: "The presence of inflammation as a necessary element has been doubted. But there can be no doubt as to the general presence of congestion or hyperplasia."

From the above extracts, which fairly represent the opinions entertained by specialists at the present time whether they adopt irritability, displacement, congestion (ordinary), or inflammation, as the prime factor in womb-disease, it is apparent that the old doctrine in regard to the morbid states of the corporeal mucous membrane, still holds sway, practice lagging behind science and following a theory dating back to the Father of medicine. Surely, physical facts proven beyond a doubt by many histologists of the highest eminence, ought to outweigh all hypothetical assumptions, however venerable from their great antiquity or dominant from their general acceptance, and practice and science to meet in accord and together unravel the tangled threads of disease. Otherwise, medicine can never make any real advance nor be anything but a paltry art, weak, uncertain and blown about by every "wind of doctrine."

It is taken as a self evident proposition at the outset that the true uterine mucous membrane is, in structure and morbid proclivities like all others lining open cavities of the body. This much being granted, it follows by the inductive method of reasoning that this membrane will be liable to the same congestive and inflammatory conditions as its fellows, will normally secrete mucus and abnormally pus, and will at times, particularly in chronic cases, require direct medication. Now, as the cervical canal has at two points an hour-glass contraction, simple physical laws will be reason enough for dilatation or incision, the only question being the choice of means. A free passage being made and the seat of the disease reached, what forbids a complete and permanent cure? Such being the argument, one would expect, seeing that specialists have so little regard for the teachings of microscopical anatomy when it chances to impugn their doctrines, that they must be so fortified by unanimity of opinion and uniformity of success as to warrant them in taking their stand on clinical facts alone. Unfortunately there seem to be no general principles, no common practice, and no satisfactory results. The utero-surgeons are driven hither and thither on an era of unrest. They are ever making trial of this or that expedient and ever racking their brains to discover something more heroic and more radical which will obviate the faults of previous operations. At last, the cavity of the uterus being laid open, and its lining membrane rasped or burned away, there is no greater novelty in reserve, unless it be the amputation of the accessible portion of the offending organ.

Here and there, however, gynæcologists of the highest eminence, who with tent, knife, and escharotic have followed this practice from its inception through all its various phases, are beginning to record their disappointment in no equivocal words.

In an address before the British Medical Association, 1873. J. Braxton Hicks says: "These researches (in regard to the uterine nerves) point out to us that we cannot ignore in our clinical pathology—indeed that we must give a prominent place to—the nerve-element. May I be permitted to make the remark that this very free inblending of nerves with the other tissues has not received—at least judging from the writings of authors—that general consideration which it deserves? Blood-vessels and exudations, mechanical pressure and obstructions are freely mentioned; and instruments are used as if the uterus were a simple elastic tissue to be dilated by main force like a piece of india rubber."

In speaking of "cutting instruments and tents used in dividing or dilating the supposed obstructing part or stricture" in dysmenorrhœal cases, Mathews Duncan writes in the Edinburg Medical Journal, May 1872: "Their use has been most unsatisfactory. So much has this been the case that, in common with those friends on whose experience and judgment I have most reliance, I have been reluctantly forced in the meantime to discontinue the use of a most promising remedy in a most painful disorder."

In a paper read before the Medical Library and Journal Association, June 5th, 1874, Thomas Addis Emmet makes certain statements, starting and significant, seeing that he, in conjuuction with Sims, has for some twenty years held the position of surgeon in chief to the Woman's Hospital of the State of New York. "In years past I have honestly overcome all obstructions, and by the aid of the knife I have opened up the uterine canal to such an extent that it was impossible for any mechanical obstacle to exist; and I did not cure my patients." "I have never seen a case permanently benefited by the operation, except in rare instances, where pregnancy fortunately took place during a slight remission of symptoms, due to the revulsive action attending the process of reparation. I can, moreover, state that I have never known the malpractice of any other surgical procedure followed at times by such evil consequences." "The various devices for forcing the uterus into an upright position to a point which the organ likely never occupied when in a healthy state, are faulty in theory and wrong in practice * * * * I deprecate even more the intra-uterine stem-pessary, for, had this instrument been the device of the Evil One himself, its use could not be productive of more danger." "Rare indeed is the necessity for applying, within the uterine canal, caustics, the cautery, or the strong mineral acids. It is true that these remedies act promptly, as far as to heal an erosion and to check all uterine discharge. But we cannot restore the patient to health by so far changing the character of the mucous membrane as to leave it a mere cicatricial surface." Regarding congestion (ordinary) as the "primary condition" in uterine disease he says: "We may look in vain, *post mortem*, for any evidence of a previously—existing endometritis, so called, and ulceration of the cervix as it is termed. As to the treatment of this congestion he has found that "in the simple remedy, hot water vaginal injections, we possess the most valuable means of relief when properly administered."

If the case stand thus with the utero-surgeons, even when judgment is rendered by themselves, it will require no other evidence to convince other members of the profession that, by a resort to knives, dilators, cauterants, and the like, a very grave mistake has been committed. The fact is, the benign diseases of the uterus are, or at least ought to be, the province of the physician alone, who, if he avail himself fully of the resources of his art, will seldom, perhaps never, need the services of the surgeon, and will more certainly restore the genital, than other organs. to a state of functional integrity.

To do this, the attendant should, instead of going counter to, act in concert with the physiological laws presiding over menstruation and pregnancy, and the menstrual and puerperal involutions; should, instead of wasting time on the effects, attack the hidden causes of disease; and should, instead of destroying, restore the original condition, and with it the normal function of each part.

Proceeding on this basis, and adopting the empirical plan, the writer, as published more than two years since in his work on the Diseases and Displacements of the Uterus and recently in articles contributed to Medical Journals, arrived at certain noteworthy conclusions, which, singularly in accord with the most recent discoveries in hysterology, may be summarized under the following heads:

1st. The menstruant female is alone liable to womb disease.

2d. The uterus, ovaries, and vagina are erectile organs.

3d. Menstruation and pregnancy are each a state of erection.

4th. Involution is the subsidence of this unique vital activity and a return to a state of rest.

5th. Womb disease is due to a faulty involution, a continuance of the nerve stimulus and blood supply in the interval.

6th. The peculiar congestion attendant upon menstruation and pregnancy and that upon benign disease of the uterus are homologous.

7th. Congestion and inflammation as observed in other organs is foreign to the genital.

8th. The corporeal mucous membrane grows, degenerates, and falls proportionately with the volume and activity of the underlying circulation.

9th. This membrane regains its normal condition with the uterine walls whence it derives its nervous and vascular supply.

10th. The sympathetic nerve which is distributed as liberally to the uterus and ovaries as to any of the abdominal viscera is the prime factor in menstruation, pregnancy and womb-disease.

11th. The symptoms of inflammation and stricture, together with all others, disappear spontaneously upon the repletion of the veins and the irritability of the nerves being reduced to the normal standard.

12th. Involution, menstrual and puerperal, being attained by a hæmorrhagic flow, the proper time of treatment is sufficiently indicated.

13th. Art should imitate Nature and follow the lead of physiological laws.

14th. The loss of blood when just enough to relieve the tension of the uterine vascular canals and promote the retraction of the uterine muscular fibres is the remedy par excellence.

15th. The result is almost always fortunate and never disastrous.

